# Supplementary material for: Large T cell clones expressing immune checkpoints increase during multiple myeloma evolution and predict treatment resistance
Source: Nat Commun. 2023 Sep 20;14:5825. doi: 10.1038/s41467-023-41562-6 (PMC10511411; doi:10.1038/s41467-023-41562-6)
Supplement: Supplementary file 1 — Supplementary Information [file 41467_2023_41562_MOESM1_ESM.pdf]

Single T cell profiles in multiple myeloma reveals dysfunction of large T cell clones and phenotypic markers of response to lenalidomide-based combinations

Cirino Botta [1,2*#], Cristina Perez [2*], Marta Larrayoz [2], Noemi Puig [3], Maria-Teresa Cedena [4], Rosalinda Termini [2], Ibai Goicoechea [2], Sara Rodriguez [2], Aintzane Zabaleta [2], Aitziber Lopez [2], Sarai Sarvide [2], Laura Blanco [2], Daniele M. Papetti [5], Marco S. Nobile [6,7], Daniela Besozzi [5,7], Massimo Gentile [8], Pierpaolo Correale [9], Sergio Siragusa [1], Albert Oriol [10], Maria Esther González-Garcia [11], Anna Sureda [12], Felipe de Arriba [13], Rafael Rios Tamayo [14], Jose-Maria Moraleda [13], Mercedes Gironella [15], Miguel T. Hernandez [16], Joan Bargay [17], Luis Palomera [18], Albert Pérez-Montaña [19], Hartmut Goldschmidt [20], Hervé Avet-Loiseau [21], Aldo Roccaro [22], Alberto Orfao [23,24], Joaquin Martinez-Lopez [4], Laura Rosiñol [25], Juan-José Lahuerta [4], Joan Blade [25], Maria-Victoria Mateos [3], Jesús F. San-Miguel [2], Jose-Angel Martinez Climent [2], Bruno Paiva [2#], the Programa Para el Estudio de la Terapéutica en Hemopatías Malignas/Grupo Español de Mieloma (PETHEMA/GEM) cooperative group and the iMMunocell study group

* Contributed equally to this study and should be considered as first authors.

#Address for correspondence:
Cirino Botta, MD, PhD. Hematology Unit, Department of Health Promotion, Mother and Child Care, Internal Medicine and Medical Specialties, University of Palermo, Via del Vespro, 90127 Palermo, Italy; e-mail: cirino.botta@unipa.it

Bruno Paiva, PhD. Clínica Universidad de Navarra; Centro de Investigación Médica Aplicada (CIMA), Av. Pío XII 55, 31008 Pamplona, Spain; e-mail: bpaiva@unav.es

1) Department of Health Promotion, Mother and Child Care, Internal Medicine and Medical Specialties, University of Palermo, Palermo, Italy; 2) Clinica Universidad de Navarra, Centro de Investigacion Medica Aplicada (CIMA), CCUN, Instituto de Investigacion Sanitaria de Navarra (IDISNA), CIBER-ONC numbers CB16/12/00369, CB16/12/00489, Pamplona, Spain; 3) Hospital Universitario de Salamanca, Instituto de Investigacion Biomedica de Salamanca (IBSAL), Centro de Investigación del Cancer (IBMCC-USAL, CSIC), CIBER-ONC number CB16/12/00233, Salamanca, Spain; 4) Hospital Universitario 12 de Octubre, CIBER-ONC number CB16/12/00369, Madrid, Spain; 5) Department of Informatics, Systems and Communication, University of Milano-Bicocca, Milan, Italy; 6) Department of Environmental Sciences, Informatics and Statistics, Ca' Foscari University of Venice, Venice, Italy; 7) Bicocca Bioinformatics, Biostatistics and Bioimaging Centre – B4, Milan, Italy; 8) Department of Oncohematology, "Annunziata" Hospital, Cosenza, Italy; 9) Medical Oncology Unit, Great Metropolitan Hospital "Riuniti" of Reggio Calabria, Reggio Calabria, Italy; 10) Institut Català d'Oncologia i Institut Josep Carreras, Badalona, Spain; 11) Servicios de Medicina Interna y Hematología, Hospital de Cabueñes, Gijón, Asturias, Spain; 12) Institut Català d'Oncologia-Hospitalet, Instituto de Investigación Biomédica de Bellvitge (IDIBELL), Barcelona, Spain; 13) Hospital Morales Meseguer, IMIB-Arrixaca, Universidad de Murcia, Murcia, Spain 14) Hospital Universitario Puerta de Hierro, Majadahonda, Spain; 15) Hospital Vall d'Hebron, Barcelona, Spain; 16) Hospital Universitario de Canarias, Santa Cruz de Tenerife, Spain; 17) Hospital Son Llatzer, Palma de Mallorca, Spain; 18) Hospital Clínico Lozano Blesa, Zaragoza, Spain; 19) Hospital Son Espases, Palma de Mallorca, Spain; 20) Department of Internal Medicine V, University of Heidelberg, Heidelberg, Germany; 21) Unite de Genomique du Myelome, IUC-T Oncopole, Toulouse, France; 22) Department of Hematology, ASST Spedali Civili di Brescia, Brescia, BS, Italy; 23) Cancer Research Center (IBMCC-CSIC/USAL-IBSAL), CIBER-ONC number CB16/12/00400, Salamanca, Spain; 24) Cytometry Service (NUCLEUS) and Department of Medicine, University of Salamanca, Salamanca, Spain; 25) Hospital Clínic IDIBAPS, Barcelona, Spain.

FIGURE S1

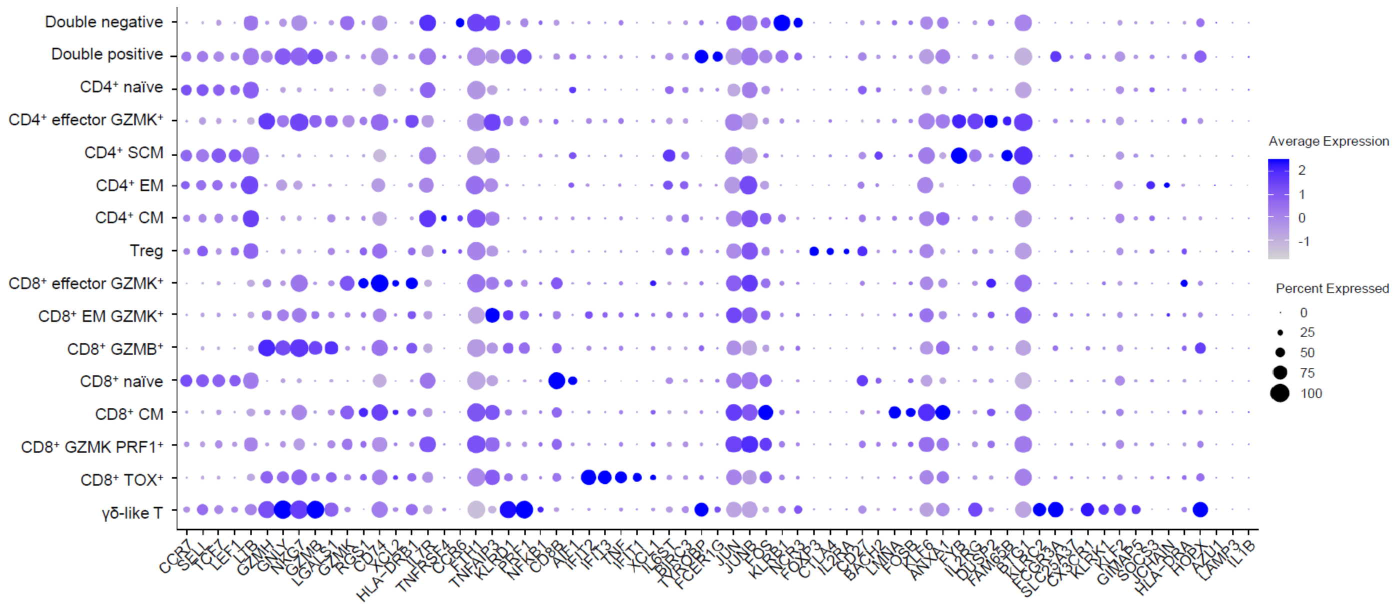

Figure S1. Genes used for the identification of T cell clusters. The 16 T cell clusters were detectable in bone marrow aspirates from healthy adults (n = 4), MGUS/SMM (n = 8) and MM (n = 10) patients. The code of each circle represents the average expression of each gene in each of the 16 clusters, whereas the size of the circle represents the percentage of cells expressing the gene.

FIGURE S2

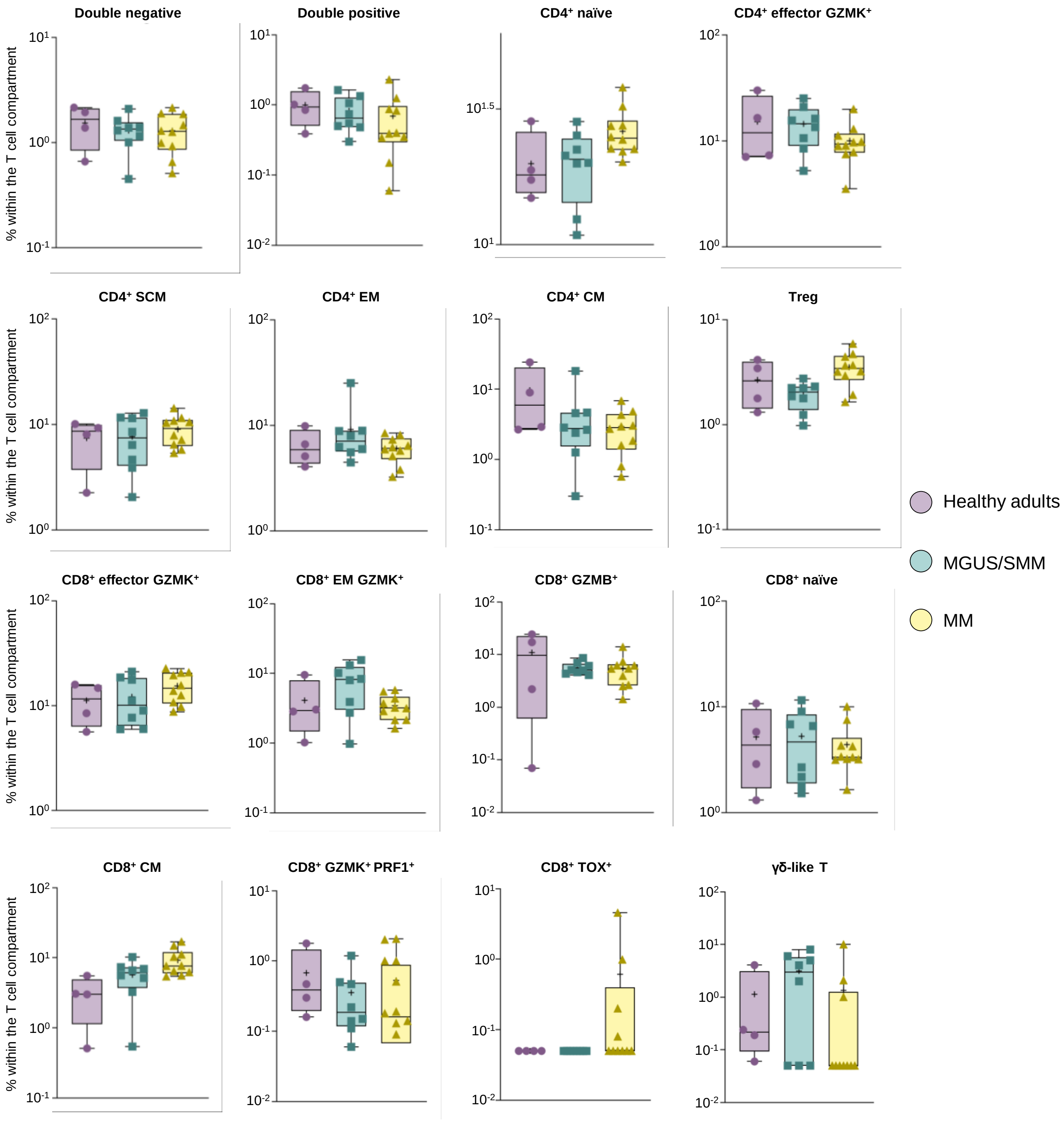

Figure S2. Abundance of T cell subsets. Box plots representing in detail the abundance of T cell subsets in each healthy adults (n = 4), MGUS/SMM (n = 8) and MM (n = 10) patients. Centre lines and error bars represent median ± minimum and maximus.

FIGURE S3

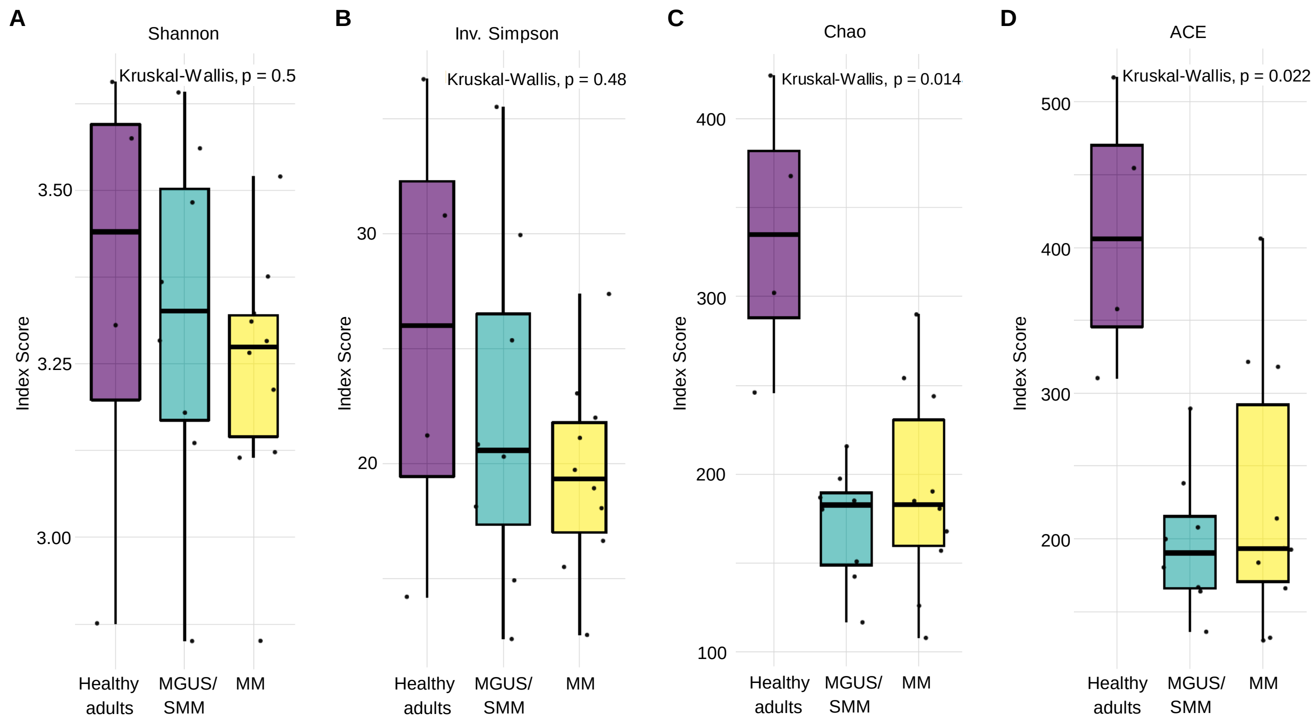

Figure S3. Diversity analysis of TCRs. TCR expansions and diversity in healthy adults (n = 4), MGUS/SMM (n = 8) and MM (n = 10) patients according to the Shannon, Inverse Simpson, Chao1 and ACE index scores. Centre lines and error bars represent mean ± standard error mean.

FIGURE S4

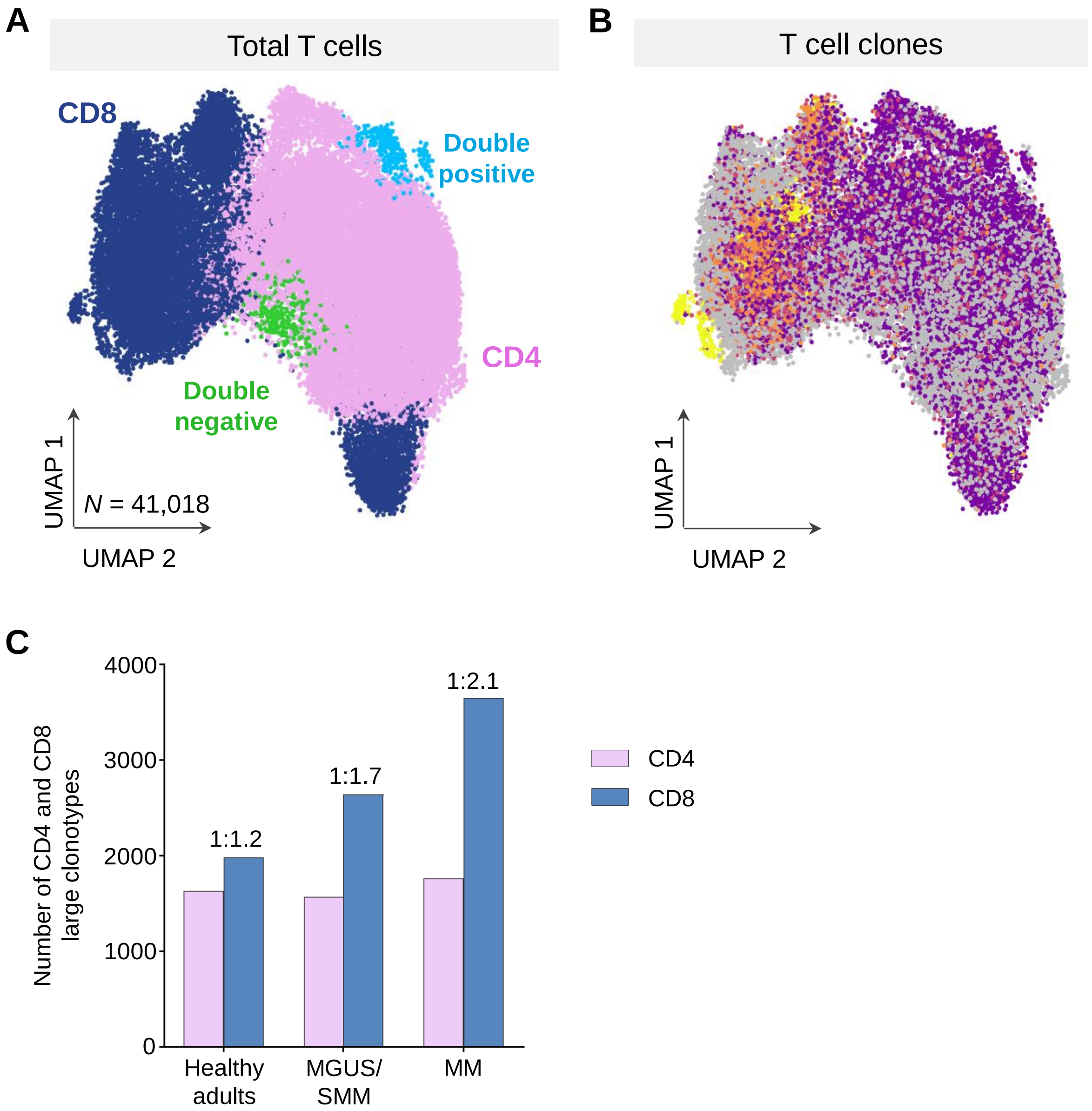

Figure S4. The T cell compartment in healthy, benign and malignant bone marrow. (A) Uniform manifold approximation and projection (UMAP) of 41,018 bone marrow T cells from healthy adults (n = 4), patients with monoclonal gammopathy of undetermined significance and smoldering multiple myeloma (MGUS/SMM, n = 8), and newly-diagnosed patients with active multiple myeloma (MM, n = 10). **(B)** UMAP of the distribution of T cell clones in bone marrow T cells from healthy adults, MGUS/SMM and MM patients, color coded according to their relative abundance (see Figure 1E). **(C)** Number and ratio of CD4 and CD8 large clonotypes in healthy adults, MGUS/SMM and MM patients. Bars represent the total number of cells with expanded clonotypes in the CD4 and CD8 compartments. Source data are provided as a Source Data file.

FIGURE S5

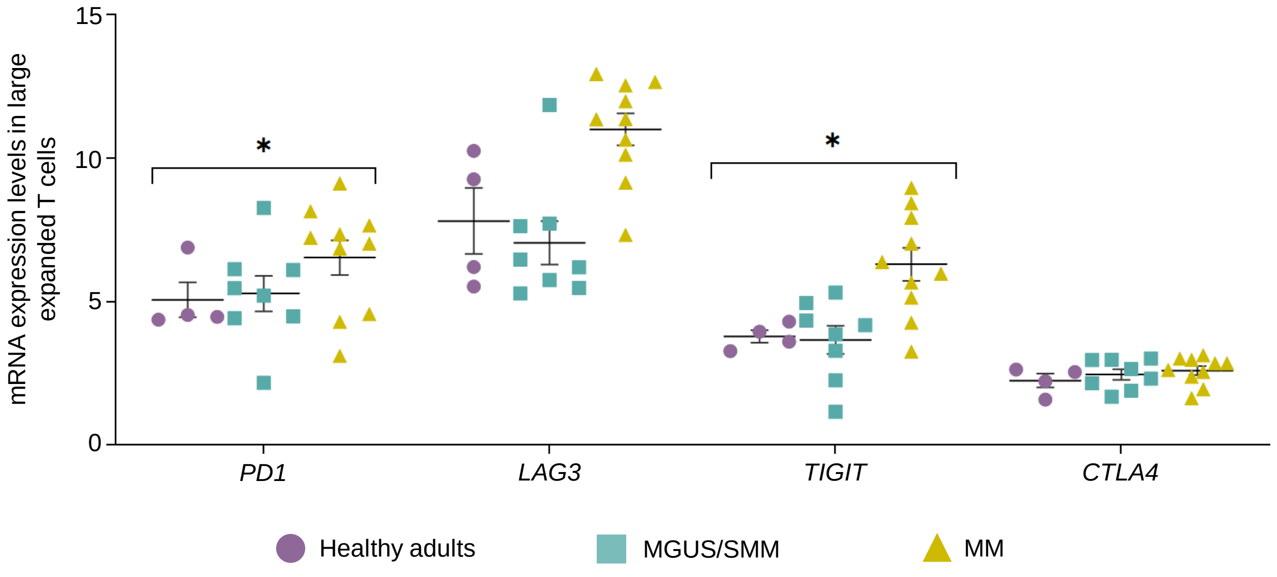

Figure S5. mRNA expression levels of immunocheckpoints. mRNA expression levels of *PD1*, *LAG3*, *TIGIT* and *CTLA4* in large expanded T cell clones from healthy adults (n = 4), MGUS/SMM (n = 8) and MM (n = 10) patients. Centre lines and error bars represent mean ± standard error mean. Dots represent each patient. *P* values were calculated using the Kruskal-Wallis test, *p = .01 and .02. Source data are provided as a Source Data file.

FIGURE S6

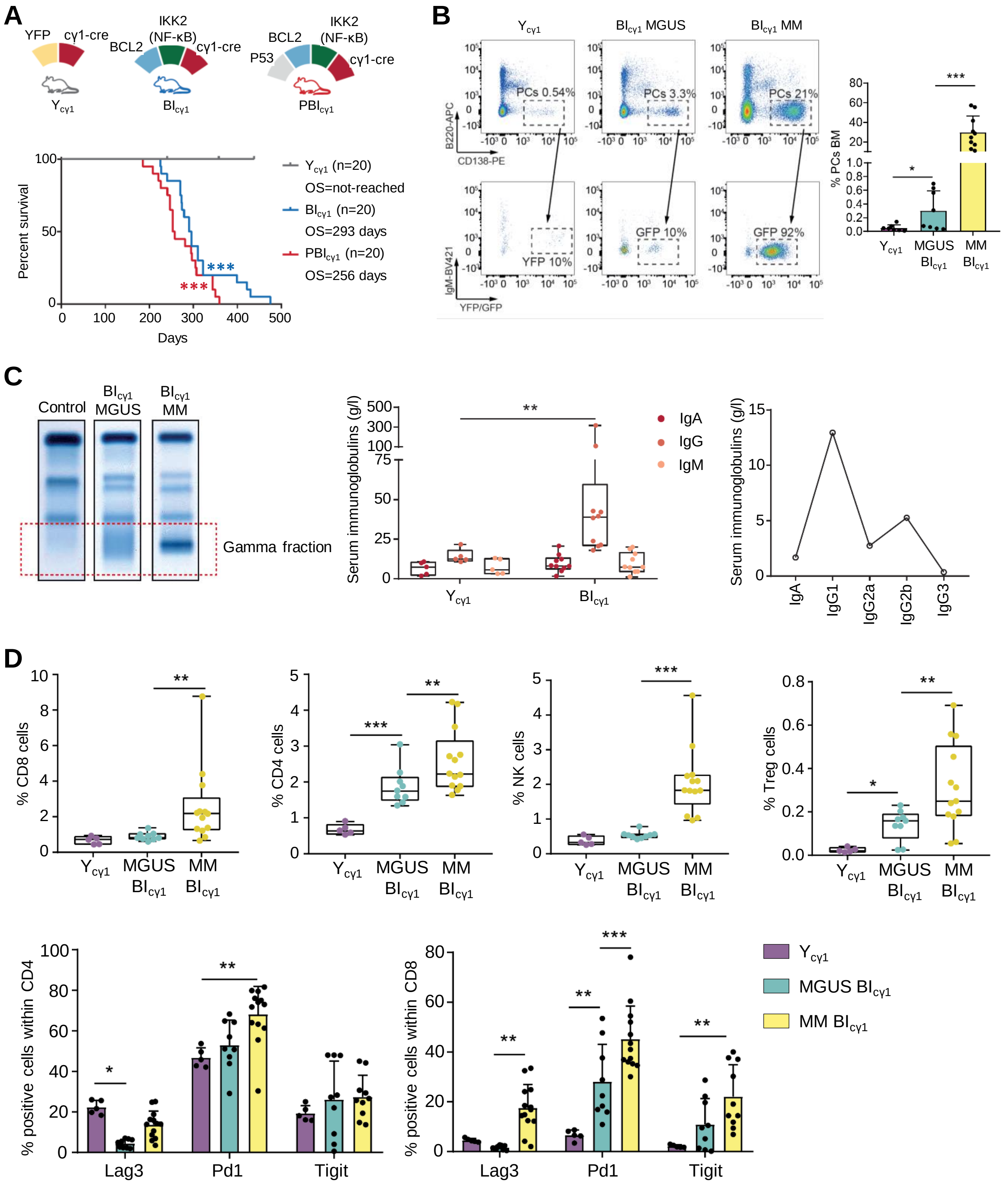

Figure S6. (A) Schematic representation of the transgenic mouse lines that develop MM. $BI_{c\gamma1}$ mice were generated by crossing mice with expression of heterozygous BCL2 and $IKK2^{NF\text{-}\kappa B}$ alleles to cγ1-cre mice. $PBI_{c\gamma1}$ mice were generated by crossing $BI_{c\gamma1}$ mice. $YFP_{c\gamma1}$ mice, generated by crossing yellow fluorescence protein reporter mice with cγ1-cre mice, were used as controls. Following immunization with red blood sheep cells, $BI_{c\gamma1}$ and $PBI_{c\gamma1}$ mice developed fully penetrant tumors in the bone marrow (BM) and shortened median overall survival (OS), as shown in the Kaplan-Meier curves. *P* values calculated using log-rank test, ***p<.001. **(B)** Flow cytometry analysis in a

representative BM sample from a $BI_{c\gamma1}$ mouse at 6 month of age (MGUS) and at the time of death (11 months, MM). Controls correspond to $YFP_{c\gamma1}$ mice. On the right, quantification of the number of transgenic PCs in the BM of $YFP_{c\gamma1}$ (n=7) and $BI_{c\gamma1}$ mice at MGUS (n=7) and MM (n=10) stages. Error bars represent mean ± standard error mean (SEM). *P* values calculated using two-sided Student t test, *p<.05; ***p<.001. **(C)** Electrophoresis analysis of Ig secretion in serum samples from $BI_{c\gamma1}$ mice at MGUS and MM stages with respect to $YFP_{c\gamma1}$ control mice; M-spikes correspond to the γ fraction (left). Samples derive from the same experiment and gels were processed in parallel. Source data are provided as a Source Data file. Quantification of Ig isotypes in serum samples by ELISA in $BI_{c\gamma1}$ (n=10) and $YFP_{c\gamma1}$ mice (n=5). Centre and error bars represent mean±SEM. *P* values calculated using two-sided Student t test, **p<.01 (middle). A representative example of the clonal of IgG1 secretion in a $BI_{c\gamma1}$ mouse with MM (right). **(D)** Distribution of lymphoid cell subpopulations measured by flow cytometry in the BM of $BI_{c\gamma1}$ mice at MGUS (n=9) and MM (n=13) stages in comparison to control age-matched $YFP_{c\gamma1}$ mice (n=5), including $CD4^+$ and $CD8^+$ T lymphocytes, regulatory T (Treg) cells, and NK cells. The percentage of BM $CD4^+$ and $CD8^+$ T cells with surface expression of Lag3, Pd1, and Tigit at MGUS and MM stages, measured by flow cytometry (bottom). Centre and error bars represent mean±SEM. *P* values calculated using two-sided Student t test, *p<.05; **p<.01; ***p<.001. (A, B, C and D) Mice between 6-12 months and of both sexes were included. (B and D) Gating strategy included in Source Data file.

FIGURE S7

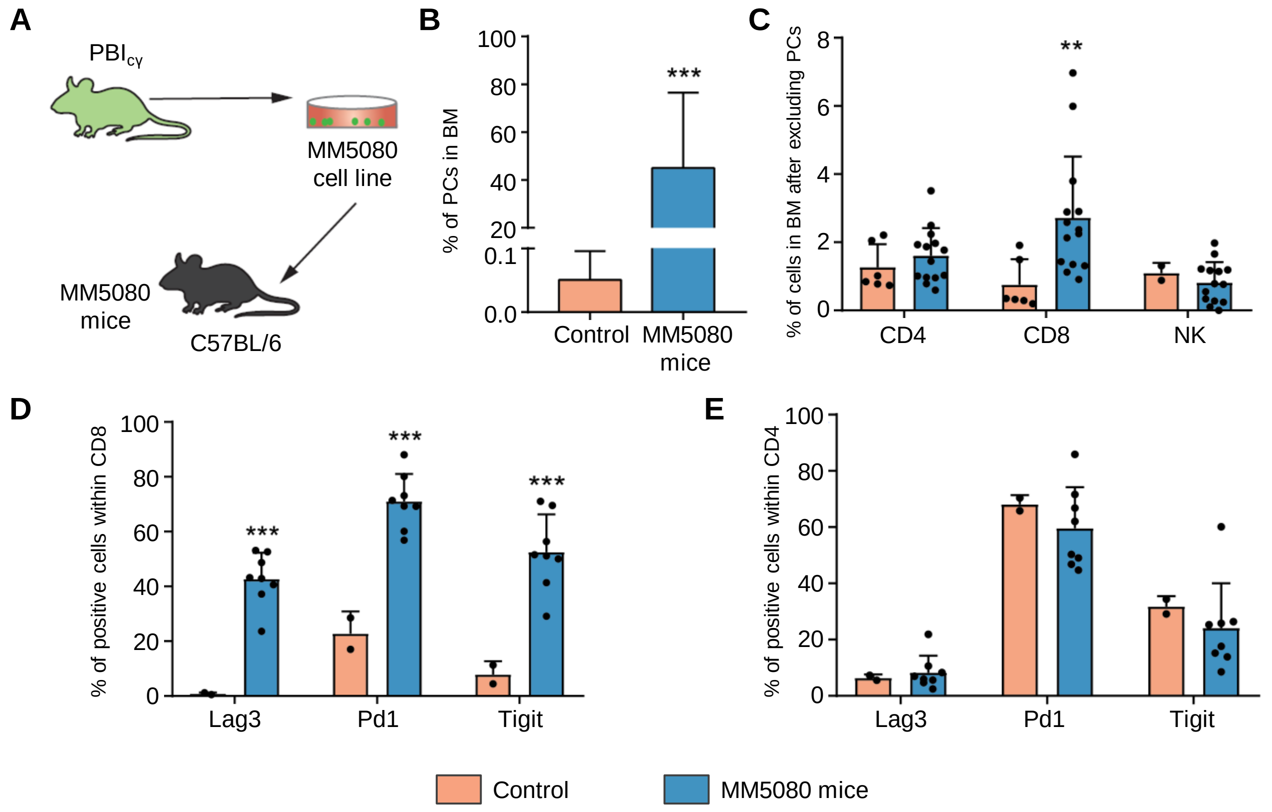

Figure S7. Cellular composition of the BM of MM5080 mice and expression of immunocheckpoints in T cells. (A) C57B6 immunocompetent mice were injected with the 5080 murine MM cell line, which was established from P53-BIcγ1 mice. **(B)** Percentage of plasma cells (PCs) in the bone marrow (BM) of control (n = 7) vs MM5080 (n = 8) mice. Error bars represent mean ± standard error mean (SEM). *P* values were calculated using the two-sided Student t test, ***p < .001. **(C)** $CD8^+$ and $CD4^+$ T cells and NK cells in the bone marrow of control (n = 6) vs MM5080 (n = 14) mice. Error bars represent mean ± SEM. *P* values were calculated using the two-sided Student t test, **p < .01. **(D)** Expression of immune checkpoints in $CD8^+$ T cells from control (n = 2) vs MM5080 (n = 8) mice. Error bars represent mean ± SEM. *P* values were calculated using the two-sided Student t test, ***p < .001. **(E)** Expression of immune checkpoints in $CD4^+$ T cells from control (n = 2) vs MM5080 (n = 8) mice. Error bars represent mean ± SEM**.** *P* values were calculated using the two-sided Student t test. (B, C, D and E) Gating strategy included in Source Data file.

FIGURE S8

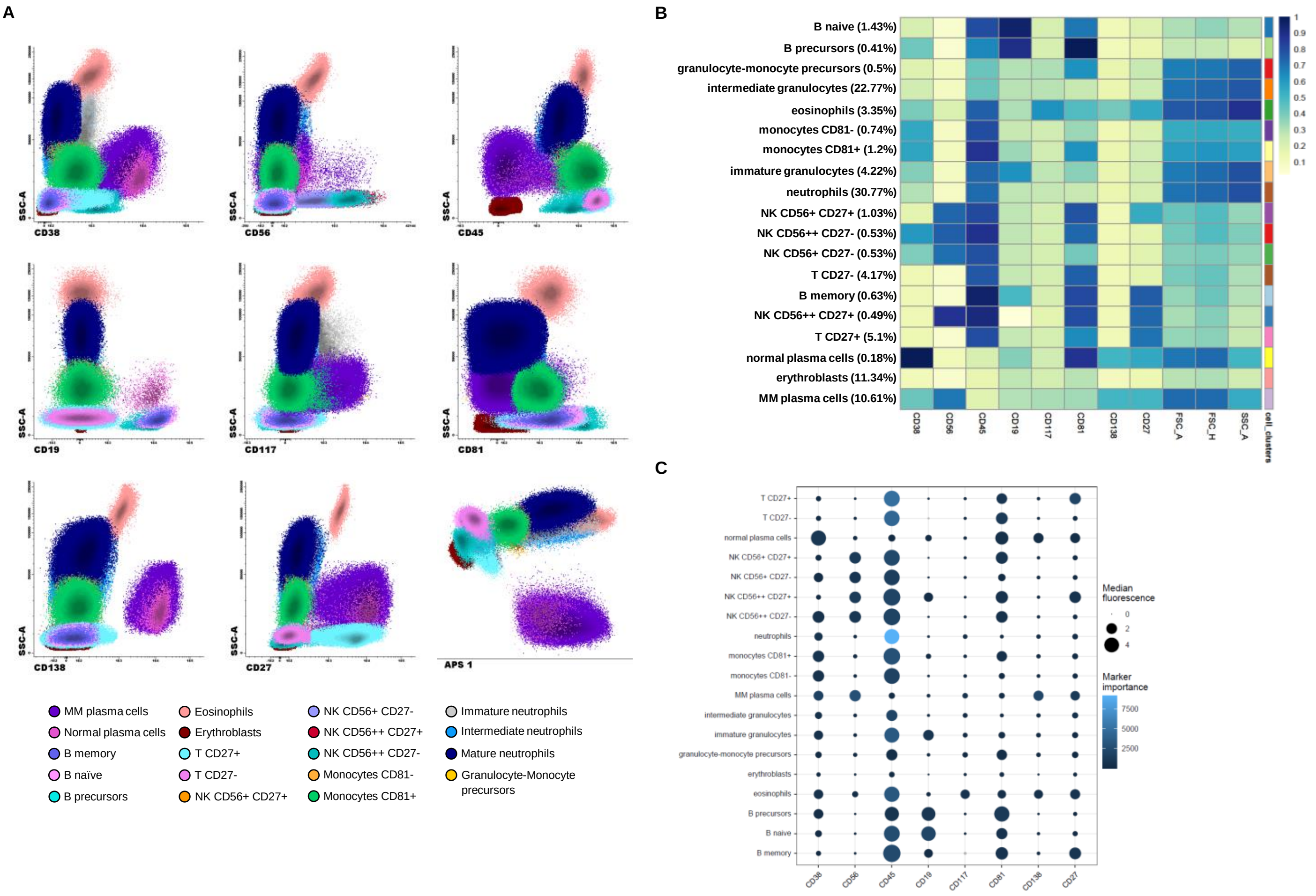

Figure S8. Identification of BM cell clusters. (A) Manual analysis of the flow cytometry data used and the corresponding identification of the 19 cell populations identified by computational analysis. (**B)** Heatmap showing the expression of all markers used for flow cytometry analysis in the 19 cell populations found in the bone marrow of MM patients. **(C)** Dotplot showing the median fluorescence of each marker used for flow cytometry analysis in the 19 cell populations.

FIGURE S9

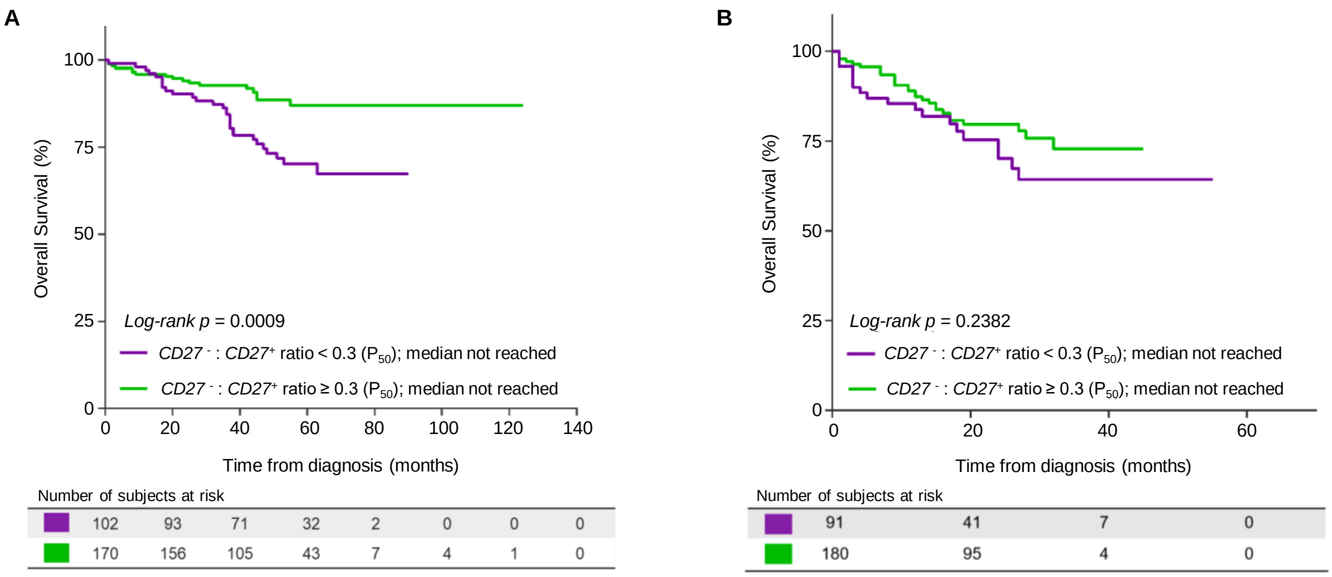

Figure S9. Overall survival of **(A)** transplant-eligible and **(B)** transplant-ineligible MM patients, stratified according to the median value of the $CD27^-$: $CD27^+$ ratio in bone marrow T cells.

FIGURE S10

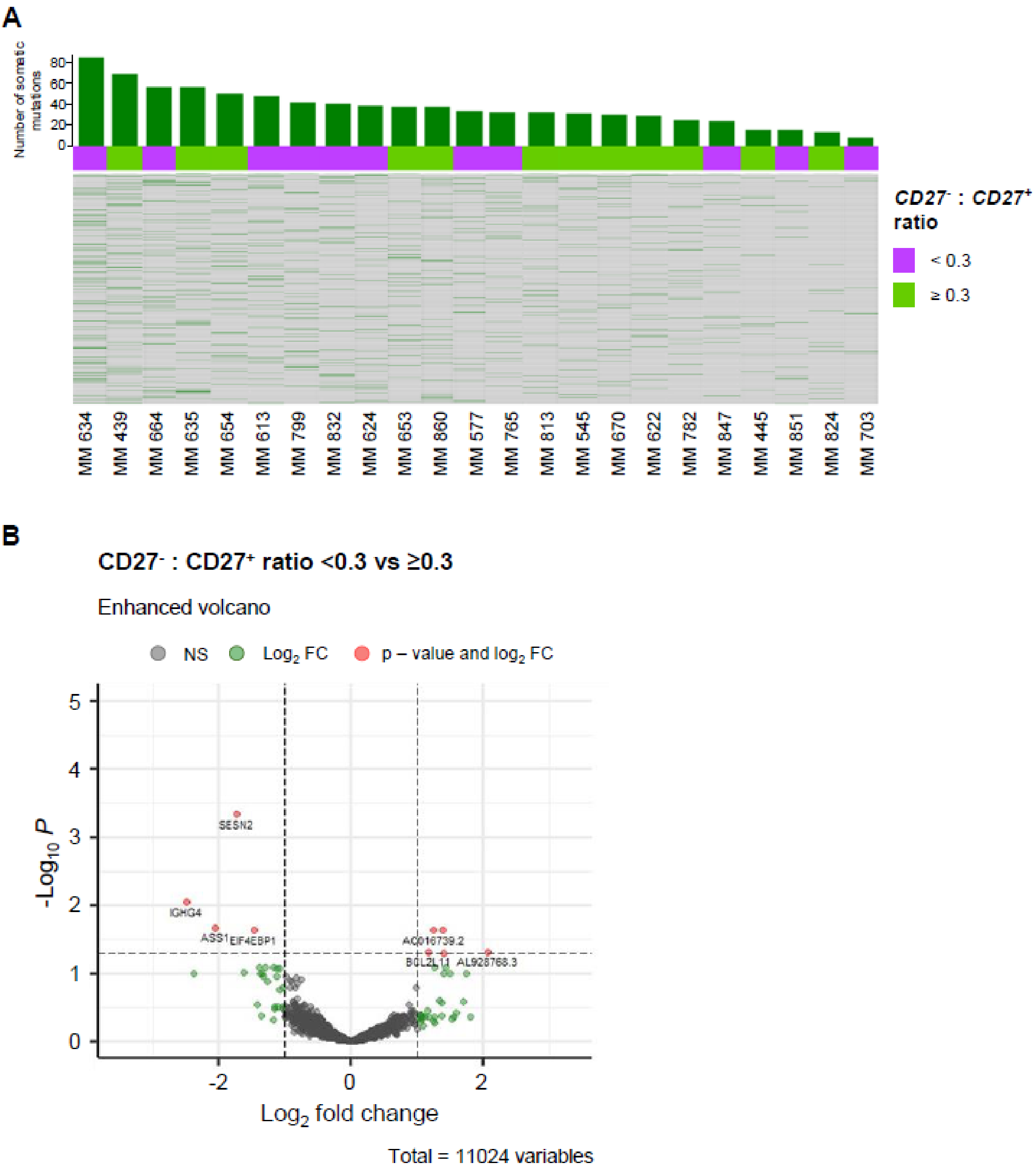

Figure S10. Analysis of the mutational burden and transcriptional profile of tumor cells. (A) Bar chart and heatmap showing the number of different mutations present in tumor plasma cells from 23 MM patients, stratified according to the median value of the $CD27^-$: $CD27^+$ ratio in bone marrow T cells. The median number of somatic mutations was 35 and 23 in the groups of patients with a median value of the $CD27^-$: $CD27^+$ ratio lower and equal or greater than 0.3, respectively. **(B)** Volcano plot representing the transcriptional profile of tumor plasma cells from MM patients, stratified according to the median value of the $CD27^-$: $CD27^+$ ratio. There were 57 differentially expressed genes between patients with a median value of the $CD27^-$: $CD27^+$ ratio lower and equal or greater than 0.3. *P* values were calculated using the Kruskal-Wallis test.

TABLE S1

Demographics and disease characteristics of the 22 individuals studied by single-cell RNA and TCR sequencing.

Patient	Health condition	Age (years)	Sex	Cytogenetic abnormalities						Cytogenetic risk
				del17p	del1p	gain1q	amp1q	IGH_FGFR3	IGH_MAF	
**Healthy1**	Healthy	43	female	N/A	N/A	N/A	N/A	N/A	N/A	N/A
**Healthy2**	Healthy	72	female	N/A	N/A	N/A	N/A	N/A	N/A	N/A
**Healthy3**	Healthy	52	N/A	N/A	N/A	N/A	N/A	N/A	N/A	N/A
**Healthy4**	Healthy	90	N/A	N/A	N/A	N/A	N/A	N/A	N/A	N/A
**MGUS/SMM1**	MGUS	68	male	N/A	N/A	N/A	N/A	N/A	N/A	N/A
**MGUS/SMM2**	MGUS	87	male	negative	negative	negative	positive	negative	negative	high risk
**MGUS/SMM3**	MGUS	67	female	negative	negative	negative	negative	negative	negative	standard risk
**MGUS/SMM4**	MGUS	55	male	negative	negative	positive	negative	negative	negative	standard risk
**MGUS/SMM5**	SMM	35	male	N/A	N/A	N/A	N/A	N/A	N/A	N/A
**MGUS/SMM6**	SMM	51	female	N/A	N/A	N/A	N/A	N/A	N/A	N/A
**MGUS/SMM7**	SMM	51	male	N/A	N/A	N/A	N/A	N/A	N/A	N/A
**MGUS/SMM8**	SMM	42	female	negative	negative	negative	negative	negative	negative	standard risk
**MM1**	NDMM	55	female	negative	negative	negative	negative	negative	negative	standard risk
**MM2**	NDMM	54	female	negative	negative	negative	negative	negative	negative	standard risk
**MM3**	NDMM	50	female	negative	negative	negative	negative	negative	negative	standard risk
**MM4**	NDMM	58	female	negative	negative	negative	negative	negative	negative	standard risk
**MM5**	NDMM	64	female	negative	negative	negative	negative	negative	negative	standard risk
**MM6**	NDMM	51	male	positive	negative	negative	negative	negative	negative	high risk
**MM7**	NDMM	71	male	negative	negative	negative	negative	negative	negative	standard risk
**MM8**	NDMM	73	female	negative	negative	negative	negative	negative	negative	standard risk
**MM9**	NDMM	66	female	negative	negative	negative	positive	negative	negative	high risk
**MM10**	NDMM	N/A	N/A	negative	negative	negative	negative	negative	negative	standard risk

TABLE S2

Assessment of the quality of the bone marrow aspirates based on the percentages of bone marrow specific cell types (B-cell precursors, mast cells and nucleated red blood cells), determined by multiparameter flow cytometry. Reference values were obtained from Puig, N. *et al*. *Cancers (Basel)* (2021).

	% within total bone marrow cells		
	**B cell precursors**	**Mast cells**	**Nucleated red blood cells**
**Healthy adults 1**	0.25	0.005	5.14
**Healthy adults 2**	0.33	0.045	1.45
**Healthy adults 3**	2.21	0.007	9.22
**MGUS/SMM 1**	0.98	0.007	9.54
**MGUS/SMM 2**	0.81	0.049	14.5
**MGUS/SMM 3**	2.15	0.009	1.23
**MM 1**	0.11	0.006	6.77
**MM 2**	0.66	0.006	1.92
**MM 3**	0.48	0.008	4.62
**MM 4**	2.76	0.047	2.57
**MM 5**	0.18	0.007	5.58
**MM 6**	0.42	0.006	7.44
**Reference values**			
Median (range)	0.35 (0.01–3.64)	0.002 (0.0002–0.05)	4.6 (0.1–15.2)

TABLE S3

Distribution of T cell subsets in bone marrow aspirates of healthy adults (n = 4), MGUS/SMM (n = 8) and MM (n = 10) patients.

	CD4 naïve	CD4 effector GZMK	CD4 SCM	CD4 EM	CD4 CM	Treg	CD8 effector GZMK	CD8 EM GZMK	CD8 GZMB	CD8 naïve	CD8 CM	CD8 GZMK PRF1	CD8 TOX	gd-like T	Double negative	Double positive
**Healthy1**	18.81	29.88	2.25	9.86	24.22	1.31	8.45	1.02	0.07	1.31	0.51	0.3	0	0.24	1.38	0.39
**Healthy2**	17.32	7.06	9.27	4.07	2.91	1.78	15.9	9.54	17.22	5.78	5.54	0.47	0	0.19	1.94	1.01
**Healthy3**	28.47	16.48	8.04	6.61	8.99	4.15	5.61	3.03	2.21	10.72	3.07	0.16	0	0.06	0.66	1.74
**Healthy4**	14.86	7.29	10.09	5.1	2.67	3.44	14.74	2.84	24.26	2.88	3	1.78	0	4.05	2.15	0.85
**MGUS/SMM1**	12.38	5.21	12.8	5.95	0.3	2.23	18.54	13.1	8.63	2.68	10.24	1.19	0	6	0.45	0.3
**MGUS/SMM2**	10.84	20.91	4.64	8.9	4.66	1.24	21.12	10.15	6.94	2.17	3.25	0.15	0	2.01	1.39	1.63
**MGUS/SMM3**	20	25.16	2.05	8.84	18.25	0.98	5.98	0.98	5.09	1.52	0.54	0.5	0	8	1.61	0.5
**MGUS/SMM4**	22.39	8.44	6.38	25.16	1.26	1.86	8.97	2.72	6.12	1.79	7.38	0.47	0	5	1	1.06
**MGUS/SMM5**	19.9	16.21	3.88	7.95	4.58	1.78	11.2	8.35	4.97	9.03	5.6	0.06	0	4	1.15	1.34
**MGUS/SMM6**	21.26	13.47	11.47	5.54	2.64	2.24	7.66	15.56	4.63	6.58	6.61	0.22	0	0	1.38	0.74
**MGUS/SMM7**	28.37	15.59	11.64	6.26	2.85	2.31	5.93	8.01	4.32	6.81	5.16	0.11	0	0	2.09	0.55
**MGUS/SMM8**	25.24	10.58	8.52	4.46	2.4	2.75	17.65	3.91	4.09	11.54	6.94	0.14	0	0	1.3	0.48
**MM1**	27.34	19.87	5.35	8.09	6.85	1.92	12.49	2.87	2.5	3.32	6.43	0.18	0	1	0.92	0.87
**MM2**	32.3	12.89	7.86	3.79	1.85	3.19	9.7	2.13	7.38	4.21	11.13	0.14	0	2.09	0.51	0.83
**MM3**	37.93	11.2	7.12	7.31	2.72	5.89	8.74	2.14	1.42	7.51	6.21	0.13	0	0	1.29	0.39
**MM4**	22.03	3.52	6.42	5.92	0.57	1.64	13.78	3.21	13.92	1.64	14.85	0.19	0.99	10	1.26	0.06
**MM5**	20.16	8.93	11.49	5.75	1.59	4.69	20.78	5.48	5.39	3.18	10.26	0.09	0	0	1.86	0.35
**MM6**	22.49	7.79	10.34	5.1	0.8	3.64	19.45	3.71	3.93	3.2	16.9	0.51	0.1	0	1.89	0.15
**MM7**	27.45	9.67	5.74	6.41	3.04	2.92	22.61	3.15	6.19	3.37	7.65	0	0	0	1.46	0.34
**MM8**	24.36	9.77	14.19	8.43	2.95	3.21	10.71	5.76	6.16	4.28	7.63	0	0.01	0	2.14	0.4
**MM9**	22.58	7.47	10.5	6.17	4.82	3.68	20.52	1.62	5.41	3.14	5.58	2.01	4.55	0.05	0.65	1.25
**MM10**	24.9	8.98	10.81	3.25	4.32	4.43	15.55	4.35	2.64	10.01	5.42	2.06	0	0	0.99	2.29

TABLE S4

Demographics and disease characteristics of the 8 mice studied by single-cell RNA and TCR sequencing.

Mouse	Health condition	Age (days)	Sex	Genotype
**Control 1**	Healthy	551	male	Yc
**Control 2**	Healthy	551	male	Yc
**MGUS 1**	MGUS	179	female	BIc
**MGUS 2**	MGUS	179	female	BIc
**MGUS 3**	MGUS	207	male	BIc
**MM 1**	MM	307	female	BIc
**MM 2**	MM	215	female	BIc
**MM 3**	MM	322	male	BIc

TABLE S5

Distribution of T cell subsets in bone marrow aspirates of control (n = 2), MGUS (n = 3) and MM (n = 3) mice.

	Control 1	Control 2	MGUS 1	MGUS 2	MGUS 3	MM 1	MM 2	MM 3
**$CD8^+$ $TIGIT^+$**	9.56	13.48	12.57	15.96	10.52	8.45	6.32	15.36
**Treg**	16.91	14.05	25.26	13.64	13.16	13.87	22.16	19.26
**$CD8^+$ $GZMB^+$ $LAG3^+$**	9.73	11.66	4.17	4.64	17.95	13.78	10.07	18.01
**$CD8^+$ $GZMK^+$**	16.01	15.81	11.22	13.21	15.99	31.42	23.65	18.29
**$CD4^+$ $LAG3^+$**	6.01	5.43	3.72	1.99	2.47	4.93	7.41	3.62
**$CD4^+$ $TIGIT^+$**	3.80	3.25	4.40	4.51	4.61	3.41	3.46	2.90
**$CD8^+$ naïve**	12.64	10.21	8.70	12.88	6.55	4.29	3.89	5.42
**$CD4^+$ SCM**	3.61	2.75	4.03	2.98	2.43	2.52	3.95	2.55
**Double positive**	1.85	2.13	1.15	2.88	1.93	5.32	3.69	1.31
**Double negative**	1.31	1.69	3.25	3.86	5.19	1.16	1.74	0.79
**$CD8^+$ CM**	6.26	7.56	6.78	11.77	3.72	2.16	2.37	3.69
**$CD4^+$ EM**	1.31	1.79	4.41	1.70	2.32	0.70	1.40	1.33
**$CD4^+$ effector**	4.76	4.57	2.93	3.40	2.32	1.95	2.77	1.65
**$CD4^+$ $PD1^+$**	1.89	2.00	4.15	3.04	2.72	4.71	5.03	2.38
**$CD4^+$ naïve**	3.30	2.57	2.75	2.62	1.61	1.03	1.60	1.16
**$CD8^+$ $GZMB^+$ $CXCR3^+$**	0.33	0.21	0.40	0.82	6.40	0.24	0.34	0.21
**$CD8^+$ SCM**	0.74	0.86	0.11	0.10	0.11	0.06	0.14	2.08

TABLE S6

Association between the CD27^{-} : CD27^{+} T cell ratio and the International Staging System (ISS) and lactate dehydrogenase (LDH). *P* values were calculated by using the Chi-square test.

Correlation between CD27 ratio and ISS

		CD27 ratio		Total
		low	high	
ISS	1	46	72	118
	2	35	65	100
	3	19	35	54
Total		100	172	272

	value	df	Significance (bilateral)
Chi-square Pearson	.455	1	.797

Correlation between CD27 ratio and LDH

		CD27 ratio		Total
		low	high	
LDH	normal	120	108	228
	high	12	32	44
Total		132	140	272

	value	df	Significance (bilateral)
Chi-square Pearson	2.374	1	.123

TABLE S7

Association between the $CD27^-$: $CD27^+$ T cell ratio and cytogenetic abnormalities. *P* values were calculated by using the Chi-square test.

Correlation between CD27 ratio and cytogenetic abnormalities

		CD27 ratio		Total
		low	high	
del17p	absence	114	138	252
	presence	12	8	20
Total		126	146	272

	value	df	Significance (bilateral)
Chi-square Pearson	.402	1	.526

		CD27 ratio		Total
		low	high	
del1p	absence	118	138	256
	presence	8	8	16
Total		126	146	272

	value	df	Significance (bilateral)
Chi-square Pearson	.022	1	.881

		CD27 ratio		Total
		low	high	
amp1q	absence	79	95	174
	presence	47	51	98
Total		126	146	272

	value	df	Significance (bilateral)
Chi-square Pearson	.042	1	.839

		CD27 ratio		Total
		low	high	
t(4;14)	absence	102	126	228
	presence	24	20	44
Total		126	146	272

	value	df	Significance (bilateral)
Chi-square Pearson	.351	1	.553

		CD27 ratio		Total
		low	high	
t(14;16)	absence	118	138	256
	presence	8	8	16
Total		126	146	272

	value	df	Significance (bilateral)
Chi-square Pearson	.022	1	.881

TABLE S8

Details of the monoclonal antibodies used along the study.

Protein	Fluorochrome	Manufacturer	Catalog	Clone	Reactivity
CD138	BV421	BD Biosciences	562935	MI15	Human
CD27	BV510	Biolegend	302836	O323	Human
CD38	FITC	Cytognos	CYT-38F2	Multi-epitope	Human
CD56	PE	Cytognos	CYT-56PE	C5.9	Human
CD45	PerCPCy5.5	Biolegend	304028	HI30	Human
CD19	PeCy7	Beckman Coulter	IM3628	J3-119	Human
CD117	APC	BD Biosciences	333233	104D2	Human
CD81	APCH7	Cytognos	CYT-81AC750	M38	Human
CD3	BV510	BD Biosciences	563109	UCHT1	Human
CD4	PE	BD Biosciences	347327	SK3	Human
AnnexinV	APC	Immunostep	ANXVDY-200T		Human
CD8	APCH7	BD Biosciences	641409	SK1	Human
B220	APC	Biolegend	103212	RA3-6B2	Mouse
CD138	PE	Biolegend	142504	281-2	Mouse
CD19	APC-Cy7	Biolegend	115530	6D5	Mouse
CD3	PE-Cy7	Biolegend	100220	17A2	Mouse
IgM	BV421	Biolegend	406518	RMM-1	Mouse
CD4	APC	Biolegend	100516	RM4-5	Mouse
CD8	BV510	Biolegend	100752	53-6.7	Mouse
NK1.1	BV421	Biolegend	108731	PK136	Mouse
CD25	BV510	Biolegend	102041	PC61	Mouse
FOXP3	PE	Invitrogen	12-5773-82	FJK-16s	Mouse
PD1	BV421	Biolegend	135218	29F.1A12	Mouse
TIGIT	PE	Biolegend	142103	1G9	Mouse
LAG3	APC	Biolegend	125209	C9B7W	Mouse
CD11b	BV510	Biolegend	101245	M1/70	Mouse
GR1	PE-Cy7	Biolegend	108416	RB6-8C5	Mouse
