# Supplementary material for: Large T cell clones expressing immune checkpoints increase during multiple myeloma evolution and predict treatment resistance
Source: Nat Commun. 2023 Sep 20;14:5825. doi: 10.1038/s41467-023-41562-6 (PMC10511411; doi:10.1038/s41467-023-41562-6)
Supplement: Supplementary file 3 — Reporting Summary [file 41467_2023_41562_MOESM3_ESM.pdf]

nature portfolio

Corresponding author(s): Bruno Paiva

Last updated by author(s): Aug 30, 2023

Reporting Summary

Nature Portfolio wishes to improve the reproducibility of the work that we publish. This form provides structure for consistency and transparency in reporting. For further information on Nature Portfolio policies, see our Editorial Policies and the Editorial Policy Checklist.

Statistics

For all statistical analyses, confirm that the following items are present in the figure legend, table legend, main text, or Methods section.

n/a	Confirmed	
☐	☒	The exact sample size (*n*) for each experimental group/condition, given as a discrete number and unit of measurement
☐	☒	A statement on whether measurements were taken from distinct samples or whether the same sample was measured repeatedly
☐	☒	The statistical test(s) used AND whether they are one- or two-sided<br>*Only common tests should be described solely by name; describe more complex techniques in the Methods section.*
☐	☒	A description of all covariates tested
☒	☐	A description of any assumptions or corrections, such as tests of normality and adjustment for multiple comparisons
☐	☒	A full description of the statistical parameters including central tendency (e.g. means) or other basic estimates (e.g. regression coefficient) AND variation (e.g. standard deviation) or associated estimates of uncertainty (e.g. confidence intervals)
☐	☒	For null hypothesis testing, the test statistic (e.g. *F*, *t*, *r*) with confidence intervals, effect sizes, degrees of freedom and *P* value noted<br>*Give P values as exact values whenever suitable.*
☒	☐	For Bayesian analysis, information on the choice of priors and Markov chain Monte Carlo settings
☒	☐	For hierarchical and complex designs, identification of the appropriate level for tests and full reporting of outcomes
☐	☒	Estimates of effect sizes (e.g. Cohen's *d*, Pearson's *r*), indicating how they were calculated

Our web collection on statistics for biologists contains articles on many of the points above.

Software and code

Policy information about availability of computer code

Data collection

No software was used for data collection.

Data analysis

scRNA-seq and scTCR-seq data from humans and mice were analyzed separately. Sample demultiplexing, alignment to the hg38 human reference genome (or the respective mice genome) and single-cell gene count was performed using the Cell Ranger Single-Cell Software Suite v.6.0 (https://www.10xgenomics.com/). Expression matrixes were analyzed with the R package seurat 4.0 (https://satijalab.org/seurat/) and cells were filtered according to < 10% mitochondrial expression and at least 200 (but less than 2500) mRNA counts per cell. Once scaled and normalized, a genelist including the most variable genes was obtained by the FindVariableFeatures function. After removing genes belonging to the immunoglobulin families (which could work as a confounding factor during clustering), the genelist was used to derive the principal component analysis (PCA) vectors for each sample. The first 100 PCA were used to align samples (batch removal) using the R package harmony v.0.1.1. The new harmonized coordinates were used to develop UMAPs (dimensionality reduction). The shared nearest neighbor (SNN) algorithm based on 50 batch-corrected dimensions was used for clustering the cells into homogeneous groups that were manually identified according to the expression of canonical genes (see Supplemental Information) obtained from curated gene-sets. A sequential subclustering strategy (which consists basically in repeating the same steps as before on a specific subpopulation) to focus on clonotypic T cells was performed. Annotation of T cell clusters was performed taking into account the expression of genes reported in Fig. S1.

T cell clones defined by their unique CDR3 of both α and β chains were obtained from Cell Ranger v.6.0 and using the scRepertoire v.1.10.1 R package. This information was added to the Seurat object to analyze the transcriptome of these cells. scRepertoire R package was also used to assess clonotype distribution as well as to investigate clonal "diversity", characterized by clones frequency, repertoire richness and convergence. To estimate clonal expansions and richness we used Shannon, Inverse Simpson, Chao and ACE indices which have been developed to deal with under sampling (i.e. 'unseen species'), and could therefore mitigate the fact that for technical reasons, only a fraction of repertoires is sequenced and analyzed.

FCS files from 553 BM aspirates from newly diagnosed MM patients were analyzed using the semi-automated algorithm named "FlowCT" v.0.0.9, which is based on the analysis of multiple files by automated cell clustering, and Infinicyt v.2.0.

Statistical analysis was performed with Graphpad v.7 and SPSS v.25.0.0

Differential gene expression across all pairwise comparisons between groups was analyzed with Deseq2 v. 1.40.2 R package followed by k-means clustering of genes in R.

Raw FASTQ files were processed using LongRanger (v2.2.2, 10xGenomics) with default parameters. Variants were annotated using the bioinformatics software HD Genome One (DREAMgenics, Oviedo, Spain), using several databases containing functional (Ensembl, CCDS, RefSeq, Pfam), populational (dbSNP, 1000 Genomes, ESP6500, ExAC) and cancer-related (COSMIC – Release 87, ICGC – Release 27) information. In addition, 9 scores from algorithms for prediction of the impact caused by non-synonymous variants on the structure and function of the protein were used (SIFT, PROVEAN, Mutation Assessor, Mutation Taster, LRT, MetaLR, MetaSVM, FATHMM and FATHMM-MKL), and 1 score (GERP++) for evolutionary conservation of the affected nucleotide. Indel realignment was performed to correct underestimated allele frequencies. Variants with a population allele frequency higher than 0.01 were excluded. Variants detected in germline DNA (i.e., T cells) were excluded. Only mutations with a coverage higher than 6 in all samples from a patient were selected. Only variants detected in a sample with a variant frequency >= 0.15, with a mutated allele count >= 4 and droplet count >= 4 were selected. Class A HLA haplotypes were identified using optiType (v1.3.3) genotyping algorithm.

For manuscripts utilizing custom algorithms or software that are central to the research but not yet described in published literature, software must be made available to editors and reviewers. We strongly encourage code deposition in a community repository (e.g. GitHub). See the Nature Portfolio guidelines for submitting code & software for further information.

Data

Policy information about availability of data

All manuscripts must include a data availability statement. This statement should provide the following information, where applicable:

- Accession codes, unique identifiers, or web links for publicly available datasets
- A description of any restrictions on data availability
- For clinical datasets or third party data, please ensure that the statement adheres to our policy

scRNAseq read data are submitted at NCBI GEO under accession GSE205393 (https://www.ncbi.nlm.nih.gov/geo/query/acc.cgi?acc=GSE205393). hg38 human and mm10 mice reference genome were obtained from https://support.10xgenomics.com/single-cell-gene-expression/software/release-notes/build

Research involving human participants, their data, or biological material

Policy information about studies with human participants or human data. See also policy information about sex, gender (identity/presentation), and sexual orientation and race, ethnicity and racism.

Reporting on sex and gender	Subjects included in the single cell RNAseq analysis: 12 females + 7 males + 3 unknown. Ages go from 35 to 90 with a median of 59.9 years.<br><br>Subjects analyzed by multidimensional flow cytometry: A total of 565 BM samples from 4 healthy adults, 4 MGUS, 4 SMM and 553 newly-diagnosed MM patients were analyzed in this study (median ages of 62, 68, 47 and 66 years, respectively).
Reporting on race, ethnicity, or other socially relevant groupings	Subjects have not been divided in any relevant groupings.
Population characteristics	None of the healthy adults, MGUS, SMM and newly-diagnosed MM patients had received previous treatment and their median age was of 61 years.
Recruitment	Bone marrow aspirates from MM patients were obtained from participants the moment they were included in the respective clinical trials. Healthy samples were obtained from volunteers. MGUS and SMM samples were obtained from patients who arrived at the laboratory to perform a screening for diagnosis and consent the use of their samples for research.
Ethics oversight	This study was approved by the Institutional Review Board of the University of Navarra (2017.022) and was conducted according to the principles of the Declaration of Helsinki. Informed consent was obtained from all participants.

Note that full information on the approval of the study protocol must also be provided in the manuscript.

Field-specific reporting

Please select the one below that is the best fit for your research. If you are not sure, read the appropriate sections before making your selection.

[x] Life sciences [ ] Behavioural & social sciences [ ] Ecological, evolutionary & environmental sciences

For a reference copy of the document with all sections, see nature.com/documents/nr-reporting-summary-flat.pdf

Life sciences study design

All studies must disclose on these points even when the disclosure is negative.

Sample size	Any special criteria was followed to determine the sample size. These were determined by the posibility of using the samples at the moment they arrived at the laboratory and the availability of the resources to perform single-cell RNAseq.
Data exclusions	No data were excluded
Replication	In vitro experiments were performed 3 times (n = 3) and all attempts at replication were successful.
Randomization	Individuals were allocated into the different groups according to their health status (healthy, MGUS, SMM, MM). In the in vitro experiments samples were used randomly.
Blinding	Blinding is no relevant for this study because individuals were distributed by their health condition. For in vitro experiments blinding was not relevant because the only information we had from the patients was that they all have MM.

Reporting for specific materials, systems and methods

We require information from authors about some types of materials, experimental systems and methods used in many studies. Here, indicate whether each material, system or method listed is relevant to your study. If you are not sure if a list item applies to your research, read the appropriate section before selecting a response.

Materials & experimental systems

n/a	Involved in the study	
☐	☒	Antibodies
☒	☐	Eukaryotic cell lines
☒	☐	Palaeontology and archaeology
☐	☒	Animals and other organisms
☐	☒	Clinical data
☒	☐	Dual use research of concern
☒	☐	Plants

Methods

n/a	Involved in the study	
☒	☐	ChIP-seq
☐	☒	Flow cytometry
☒	☐	MRI-based neuroimaging

Antibodies

Antibodies used

Human antibodies
CD138 BV421 BD Biosciences Cat# 562935 Clone MI15
CD27 BV510 Biolegend Cat# 302836 Clone O323
CD38 FITC Cytognos Cat# CYT-38F2 Clone Multi-epitope
CD56 PE Cytognos Cat# CYT-56PE Clone C5.9
CD45 PerCPCy5.5 Biolegend Cat# 304028 Clone HI30
CD19 PeCy7 Beckman Coulter Cat# IM3628 Clone J3-119
CD117 APC BD Biosciences Cat# 333233 Clone 104D2
CD81 APCH7 Cytognos Cat# CYT-81AC750 Clone M38
CD3 BV510 BD Biosciences Cat# 563109 Clone UCHT1
CD4 PE BD Biosciences Cat# 347327 Clone SK3
AnnexinV APC Immunostep Cat# ANXVDY-200T
CD8 APCH7 BD Biosciences Cat# 641409 Clone SK1

Mouse antibodies
B220 APC Biolegend Cat#103212 Clone RA3-6B2
CD138 PE Biolegend Cat#142504 Clone 281-2
CD19 APC-Cy7 Biolegend Cat#115530 Clone 6D5
CD3 PE-Cy7 Biolegend Cat#100220 Clone 17A2
IgM BV421 Biolegend Cat#406518 Clone RMM-1
CD4 APC Biolegend Cat#100516 Clone RM4-5
CD8 BV510 Biolegend Cat#100752 Clone 53-6.7
NK1.1 BV421 Biolegend Cat#108731 PK136
CD25 BV510 Biolegend Cat#102041 Clone PC61
FOXP3 PE Invitrogen Cat#12-5773-82 Clone FJK-16s
PD1 BV421 Biolegend Cat#135218 Clone 29F.1A12
TIGIT PE Biolegend Cat#142103 Clone 1G9
LAG3 APC Biolegend Cat#125209 Clone C9B7W
CD11b BV510 Biolegend Cat#101245 Clone M1/70

	GR1 PE-Cy7 Biolegend Cat#108416 Clone RB6-8C5<br><br>No dilutions were performed. Quantity of the antibodies were determined by manufacturer's recommendations.
Validation	All antibodies were validated by the Euroflow guidelines found in https://euroflow.org/protocols/ and by each manufacturer as they show in the especific website for each antibody.

Animals and other research organisms

Policy information about studies involving animals; ARRIVE guidelines recommended for reporting animal research, and Sex and Gender in Research

Laboratory animals	The following commercial mouse strains were used in this study:<br>B6(Cg)-Gt(ROSA)26Sortm4(Ikbkb)Rsky/J; The Jackson Laboratory; Strain code:008242<br>B6.Cg-Tg(BCL2)22Wehi/J; The Jackson Laboratory; Strain code:002319<br>B6.129P2(Cg)-Ighg1tm1(cre)Cgn/J; The Jackson Laboratory; Strain code:010611<br>B6.129X1-Gt(ROSA)26Sortm1(EYFP)Cos/J; The Jackson Laboratory; Strain code:006148<br>C57BL/6JOlaHsd; Envigo; Strain code:057C<br><br>Transgenic mice were analyzed between 4 to 12 months-old. Experiments in the syngeneic mouse models were performed in mice between 8-12 weeks-old. Mice of both sexes were used in the study. Mice were kept under specific pathogen-free conditions and light/dark cycles of 12h. The temperature was constantly maintained between 18-23ºC.
Wild animals	This study did not involve wild animals
Reporting on sex	Mice from both sexes were used (4 females and 4 males)
Field-collected samples	This study did not involve samples collected in the field
Ethics oversight	Animals used in this study were kept under specific pathogen-free conditions in the animal facilities of the Center for Applied Medical Research CIMA at the University of Navarra. Animal experimentation was approved by the Ethical Committee of Animal Experimentation of the University of Navarra and by the Health Department of the Navarra Government.

Note that full information on the approval of the study protocol must also be provided in the manuscript.

Clinical data

Policy information about clinical studies

All manuscripts should comply with the ICMJEguidelines for publication of clinical research and a completedCONSORT checklist must be included with all submissions.

Clinical trial registration	PETHEMA/GEM2012MENOS65: #NCT01916252<br>PETHEMA/GEMCLARIDEX: # NCT02575144
Study protocol	PETHEMA/GEM2012MENOS65: This protocol is a national, multicenter, comparative, open-label, randomized trial comparing the progression free survival (PFS) of two pre-transplant conditioning regimens (BUMEL versus. MEL-200).<br>A total of 460 patients will be enrolled in the study. Scheduled evaluations and study visits will take place during the pre-treatment, treatment and follow-up periods.<br>The pre-treatment period includes the screening visit in which participants provide informed consent in writing in order to take part in the study. The patient is then assessed to determine his/her eligibility. The selection process will begin 21 days before the first dose of medication is administered (days -21 to 0). During the treatment period, eligible patients will be included in the study and given six cycles of induction treatment with bortezomib/ lenalidomide / dexamethasone (VRD-GEM). Each cycle will last 28 days, during which SC bortezomib will be administered on days 1, 4, 8 and 11, oral lenalidomide on days 1-21 of each cycle, and oral dexamethasone on days 1-4 and 9-12 of the cycle.<br>After the first three induction cycles, and in the absence of progression or unacceptable toxicity, peripheral blood hematopoietic stem cells will be mobilized and collected using G-CSF for later autologous transplantation. Patients will be randomized in a 1:1 allocation ratio to receive conditioning treatment with MEL-200 versus BUMEL. Randomization will take place at the beginning of the study, once the screening is complete and the patient's eligibility verified. Three months after transplantation, patients will receive two cycles of consolidation treatment with VRD-GEM at the same doses administered during induction treatment.<br>Once the treatment phase is complete, patients will begin the follow-up phase in which they will be visited every three months to evaluate disease progression and survival.<br>The full clinical trial can be accessed at: https://clinicaltrials.gov/study/NCT01916252<br><br>PETHEMA/GEMCLARIDEX: This phase III study, open-label, randomized study investigating lenalidomide and dexamethasone with and without biaxin in subjects with newly diagnosed, previously untreated MM. Eligible subjects will be randomized in a 1:1 ratio to receive a regimen consisting of either biaxin, lenalidomide, and low-dose dexamethasone (BiRd arm), or lenalidomide and low-dose dexamethasone (Rd arm). 306 patients will be included (50% in Spain (153) and 50% in the USA (153).<br>The full clinical trial can be accessed at: https://clinicaltrials.gov/study/NCT02575144
Data collection	PETHEMA/GEM2012MENOS65: Data collection is performed at diagnosis, after induction treatment, at day 100 after ASCT and after consolidation treatment.<br>Hospitals PETHEMA/GEM2012MENOS65: Hospital Durán i Reynals - ICO L´Hospitalet, H. Althaia, Xarxa Asistencial de Manresa,

Hospital Esp. de Jerez de la Frontera, Hospital Son Espases (Son Dureta), Hospital Son Llátzer, Hospital de Gran Canaria Dr. Negrín, Hospital Nuestra Señora del Prado, Complejo Universitario de Toledo, Hospital General de Albacete, Hospital Univ. Fundación de Alcorcón, Hospital General Univ. de Alicante, Hospital Torrevieja Salud UTE, Hospital del Tajo, Hospital German Trias i Pujol, Hospital de Cruces, Hospital Clinic i Provincial de Barcelona, Hospital de la Santa Creu i Sant Pau, Hospital del Mar, Hospital Vall d´Hebrón, Hospital Universitario de Burgos, Hospital General Univ. Santa Lucía, Hospital General de Castellón, Hospital General de Ciudad Real, Hospital San Pedro de Alcántara (Complejo Hospitalario de Cáceres), Hospital del Vinalopó, Hospital de Fuenlabrada, Hospital de Cabueñes, H. Univ. de Girona Dr. Josep Trueta (ICO), Complejo Hosp. Virgen de las Nieves, Hospital Universitario de Guadalajara, Hospital Severo Ochoa, Hospital de León, Hospital Universitari Arnau de Vilanova de Lleida, Hospital San Pedro, Centro Oncológico MD Anderson, Fundación Jiménez Díaz-UTE, Hospital Clínico Universitario San Carlos, Hospital General Univ. Gregorio Marañón, Hospital Infanta Cristina, Hospital Infanta Leonor, Hospital Infanta Sofía, Hospital Ramón y Cajal, Hospital Universitario 12 de Octubre, Hospital Universitario de la Princesa, Hospital Universitario La Paz, Hospital Universitario Madrid Sanchinarro, Hospital Universitario Puerta de Hierro-Majadahonda, Complejo Hospitalario Costa del Sol, Hospital Morales Meseguer, Hospital Universitario Virgen de la Arrixaca, Complejo Hospitalario Ourense, Hospital Universitario Central Asturias, Clínica Universidad de Navarra, Complejo Hospitalario de Navarra (Hospital Virgen del Camino), Complejo Hospitalario Pontevedra, Hospital de Sabadell (Parc Taulí), Hospital Clínico de Salamanca, Hospital Universitario de Donostia, Hospital Univ. Marqués de Valdecilla, Complejo Universitario de Santiago, Hospital General de Segovia, Complejo Hosp. Regional Virgen del Rocío, Hospital Nuestra Señora de Valme, Hospital Santa Bárbara, H. Universitari de Tarragona Joan XXIII, Hospital Universitari Mutua de Terrassa, Hospital Clínico Universitario de Valencia, Hospital Universitario Dr. Peset, Hospital Universitario La Fe, Hospital Clínico de Valladolid, Hospital Universitario Río Hortega, Hospital de Txagorritxu, Hospital Clínico Universitario Lozano Blesa, Hospital Miguel Servet

PETHEMA/GEMCLARIDEX: Data collection was performed at diagnosis, after induction treatment and after each 28-day cycle.
Hospitals PETHEMA/GEMCLARIDEX: CHUAC, Hospital Universitario Germans Trias i Pujol, Hospital Clinic, Hospital Universitario Vall d'Hebron, Hospital General de Castelló, Hospital de Cabueñes, Hospital Universitario Virgen de las Nieves, H. del SAS de Jerez, Hospital de León, H. U. Gregorio Marañón, Hospital Universitario 12 de Octubre, Hospital Universitario de la Princesa, Hospital Costa del Sol, Hospital General Universitario Morales Meseguer, Hospital Universitario Virgen de la Victoria, Complejo Hospitalario de Navarra, Hospital Univeristario Salamanca, Hospital Marqués de Valdecilla, Hospital Universitario de Santiago de Compostela, Hospital Universitario Virgen de Valme, Hospital Universitario Virgen del Rocío, Hospital Universitario de Canarias, H. Clínico de Valencia, Hospital Universitario Dr Peset, Hospital Universitario y Politécnico La Fe, H. U. Txagorritxu

Outcomes

PETHEMA/GEM2012MENOS65: Primary Outcome Measures: Progression Free Survival to measure the treatment efficacy [Time Frame: 2 years]
Secondary Outcome Measures :
Complete response rates to measure the treatment efficacy [Time Frame: 1 year]
Evaluation of minimal residual disease immunofixation negative-CR after each phase of treatment [Time Frame: 1 year]
Overall survival [Time Frame: Time to death]
Describe the adverse events to evaluate the safety and tolerability [Time Frame: 4 years]
To asses the outcomes, patients are studied with the extraction of bone marrow and peripheral blood for the analysis of their clinical data and the amount of pathologic plasma cells in both tissues by morphology and flow cytometry.

PETHEMA/GEMCLARIDEX: Primary Outcome Measures: Progression free survival [Time Frame: Throught the study. Approximately 4 years]
To asses the outcome, patients are studied with the extraction of bone marrow and peripheral blood for the analysis of their clinical data and the amount of pathologic plasma cells in both tissues by morphology and flow cytometry.

Flow Cytometry

Plots

Confirm that:

- [x] The axis labels state the marker and fluorochrome used (e.g. CD4-FITC).
- [x] The axis scales are clearly visible. Include numbers along axes only for bottom left plot of group (a 'group' is an analysis of identical markers).
- [x] All plots are contour plots with outliers or pseudocolor plots.
- [x] A numerical value for number of cells or percentage (with statistics) is provided.

Methodology

Sample preparation

In all cases, samples were stained following the EuroFlow lyse, wash and stain standard sample preparation protocol, adjusted to 10^6 nucleated cells. EDTA-anticoagulated human bone marrow aspirates were stained with the following combination of the monoclonal antibodies: CD138-BV421, CD27-BV510, CD38-FITC, CD56-PE, CD45-PerCPCy5.5, CD19-PECy7, CD117-APC and CD81-APCH7. Bone marrow cells from mice were labelled with B220 (RA3-6B2), CD3 (17A2), NK-1.1 (PK136), CD11b (M1/70) and Gr-1 (RB6-8C5), all from Biolegend (San Diego, CA).

Instrument

Data acquisition was performed in a FACSCanto II flow cytometer (Becton Dickinson/BD Biosciences, San Jose, CA).

Software

FACSDiva 6.1 sofware (BD Biosciences) and Infinicyt v2.0 (Cytognos SL, Salamanca, Spain).

Cell population abundance

Approximatelly, neutrophils represent 60%, monocytes 20% and lymphocytes 30% of bone marrow and peripheral blood samples.

Gating strategy

Gates were performed selecting lymphocytes, monocytes, neutrophils by FSC/SSC and expression of CD45. Subsets of these

populations were gated using the information provided by the rest of the markers available in each tube.

- [x] Tick this box to confirm that a figure exemplifying the gating strategy is provided in the Supplementary Information.
