# Supplementary material for: Large T cell clones expressing immune checkpoints increase during multiple myeloma evolution and predict treatment resistance
Source: Nat Commun. 2023 Sep 20;14:5825. doi: 10.1038/s41467-023-41562-6 (PMC10511411; doi:10.1038/s41467-023-41562-6)
Supplement: Supplementary file 4 — Description of Additional Supplementary Files [file 41467_2023_41562_MOESM4_ESM.pdf]

Description of Additional Supplementary Files

Supplementary Dataset 1.
Description: Gene signatures used for the identification of T cell clusters in bone marrow aspirates of healthy adults (n = 4), MGUS/SMM (n = 8) and MM (n = 10) patients. P values were calculated using the Kruskal-Wallis test.

Supplementary Dataset 2.
Description: Transcriptional phenotype of small, medium and large T cell clones in bone marrow aspirates from healthy adults (n = 4), MGUS/SMM (n = 8) and MM patients (n = 10).

Supplementary Dataset 3.
Description: Distribution of T cell clusters among small, medium and large T cell clones in bone marrow aspirates from healthy adults (n = 4), MGUS/SMM (n = 8) and MM patients (n = 10).

Supplementary Dataset 4.
Description: Distribution of T cell clusters among small, medium and large T cell clones in bone marrow aspirates from control (n = 2), MGUS (n = 3) and MM (n = 3) mice.

Supplementary Dataset 5.
Description: Differentially expressed genes between small, medium and large T cell clones from healthy adults (n = 4), MGUS/SMM (n = 8) and MM (n = 10) patients. P values were calculated using the Kruskal-Wallis test.
